# Activation, Amplification, and Ablation as Dynamic Mechanisms of Dendritic Cell Maturation

**DOI:** 10.3390/biology12050716

**Published:** 2023-05-14

**Authors:** Jessica Bourque, Daniel Hawiger

**Affiliations:** Department of Molecular Microbiology and Immunology, Saint Louis University School of Medicine, St. Louis, MO 63104, USA

**Keywords:** dendritic cells, maturation, tolerance, immunity

## Abstract

**Simple Summary:**

The maturation of dendritic cells, potent antigen-presenting cells of the immune system, determines the fundamental ability of these cells to initiate specific responses by T cells, which are key effector cells of the adaptive immune system. Maturation is a dynamic process that is highly sensitive to specific stimuli. As the individual cellular and molecular pathways contributing to maturation are still being uncovered, an in-depth understanding of how these various mechanisms are integrated to produce specific functional outcomes remains elusive. Here, we discuss the emerging paradigm encompassing several biologically distinct mechanisms that are functionally integrated into the process of maturation.

**Abstract:**

T cell responses to cognate antigens crucially depend on the specific functionality of dendritic cells (DCs) activated in a process referred to as maturation. Maturation was initially described as alterations of the functional status of DCs in direct response to multiple extrinsic innate signals derived from foreign organisms. More recent studies, conducted mainly in mice, revealed an intricate network of intrinsic signals dependent on cytokines and various immunomodulatory pathways facilitating communication between individual DCs and other cells for the orchestration of specific maturation outcomes. These signals selectively amplify the initial activation of DCs mediated by innate factors and dynamically shape DC functionalities by ablating DCs with specific functions. Here, we discuss the effects of the initial activation of DCs that crucially includes the production of cytokine intermediaries to collectively achieve amplification of the maturation process and further precise sculpting of the functional landscapes among DCs. By emphasizing the interconnectedness of the intracellular and intercellular mechanisms, we reveal activation, amplification, and ablation as the mechanistically integrated components of the DC maturation process.

## 1. Introduction

Dendritic cells (DCs), first discovered over 50 years ago by Ralph Steinman and Zanvil Cohn, are antigen-presenting cells (APCs) that are vital for the initiation and regulation of T cell responses to foreign and self-antigens. DCs are the crucial connection between the innate immune responses mediated by germline-encoded pattern recognition receptors (PRRs) and the adaptive immune responses induced by antigen-specific receptors that undergo gene rearrangement [1,2,3]. In both mice and humans, DCs comprise two main populations: conventional DCs (cDCs) and plasmacytoid DCs (pDCs) [4,5,6]. cDCs are additionally divided into the cDC1 and cDC2 subsets as defined by the transcription factors required for their development. The cDC1 subset requires the transcription factors Irf8, Id2, and Batf3 for development and can be distinguished by the expression of XCR1 in both mice and humans. In addition, some cDC1s also express B and T lymphocyte associated/attenuator (BTLA). Conversely, the cDC2 subset depends on the transcription factor Irf4 for development and is distinguished by the expression of CD172a (SIRPα) in mice [5,6,7]. While many of these markers overlap with the subset-specific expression in humans, the human cDC1 and cDC2 subsets are commonly distinguished by their expression of CD141 (BDCA3) and CD1c, respectively [4]. In the steady state, defined as the absence of specific acute pro-inflammatory stimuli, DCs generally promote T cell tolerance that largely relies on the differentiation of peripheral regulatory T (pTreg) cells [8,9,10]. While the developmental classification of cDC subsets does not strictly correspond with their distinctive immune functions, the specific subsets are characterized by a degree of functional specialization. cDC2s can efficiently promote Th2, Th17, and follicular helper T cell differentiation, whereas cDC1s have important roles in the induction of CD4^+^CD25^+^Foxp3^+^ pTreg cells, the priming of Th1 cells, and the cross-priming of CD8^+^ T cells [5,11,12,13,14]. This functional specialization is achieved through multiple mechanisms, including the expression of specific immunomodulatory molecules on their surface as well as through the production of specific cytokines under various immune conditions to promote specific T cell activation [11].

Human and murine cDCs develop from progenitors in the bone marrow that, upon dissemination to secondary lymphoid organs such as the spleen and lymph nodes (LNs), form a network of systemic cDCs that constantly interact with T cells [4,5,6]. Furthermore, some systemic cDCs are seeded throughout various organs and tissues to then migrate to LNs [15,16]. Under homeostatic conditions, such systemic cDCs are not directly exposed to extrinsic signals from microorganisms. In contrast, the cDCs found at anatomical barriers and their associated lymphoid tissues are constantly exposed to microbiota and other environmental factors. Instead, systemic cDCs, the focus of this review, constantly survey local and circulating antigens derived from parenchymal, interstitial, and other non-barrier tissues. Furthermore, systemic cDCs can present antigens from pathogens during infections as well as those introduced by intramuscular vaccinations [17,18,19,20]. While human and murine cDCs share many biological similarities, here, we concentrate mainly on studies conducted on murine cDCs due to the ability to experimentally dissect molecular mechanisms in mice.

## 2. Achieving Functional Competence through Maturation

After their original development from their bone marrow precursors, cDCs have a short life span compared to other immune cells in vivo, not exceeding several days [21]. Nevertheless, during this limited time window, cDCs can undergo an archetypical pro-immunogenic process generally referred to as “maturation”, which is triggered by canonical pathogen-associated molecular patterns (PAMPs), such as lipopolysaccharide (LPS) [15,20,22,23,24,25]. These maturation signals mediated through PRRs that are present in cDCs are required for efficient immunogenic priming of effector T cells [2,23,26,27,28]. However, the attainment of gene expression patterns that are similar to the ones observed throughout pro-immunogenic maturation may also be induced in cDCs after their complete development from bone marrow progenitors as a result of various homeostatic processes observed both in vitro and in vivo among murine cDCs [29,30]. As opposed to the well-established priming of T cells in pro-inflammatory environments, cDCs were initially characterized as having inert immune functions and were unable to prime T cells in the steady state [31]. Nevertheless, ensuing experiments unveiled specific differentiation pathways that are induced in a portion of CD4^+^ T cells upon activation in the steady state, including their conversion into Foxp3^+^ pTreg cells that exert dominant negative regulation of various immune responses and can block specific autoimmune responses [8,9,32]. Such tolerogenic functions in the steady state are inherently present in cDCs after their development from bone marrow precursors. Specifically, the expression of pro-tolerogenic molecules, including BTLA and T-cell immunoglobulin mucin-3 (TIM-3), is acquired by some cDC1s as part of their pre-determined developmental program in the steady state [33,34], resulting in a distinct population of cDCs with inherently tolerogenic functions [17,20]. Additionally, specific tolerogenic competence of cDCs might also be acquired through various homeostatic processes affecting cDCs after they have completed their initial developmental process [29,30].

In addition to such inherently tolerogenic functions of cDCs in the steady state, some microbial as well as endogenous specific maturation stimuli (including tumor-associated maturation stimuli) can also induce certain tolerogenic properties in cDCs, including those cDCs that migrate to the lymph nodes (LNs) and have important roles in maintaining homeostasis at anatomical barriers [17,35,36,37,38,39]. It still remains to be determined if these cells expand existing Treg cells, induce pTreg cells de novo, or modify other T cells’ activation in order to induce tolerance in antigen-activated T cells [17,40,41,42,43,44,45,46,47,48]. Furthermore, cDCs with induced tolerogenic functions may also limit anti-tumor immune responses [39,49]. Overall, various extrinsic factors may have different pro-immunogenic or pro-tolerogenic impacts, underscoring the importance of signaling networks that underlie the broader cDC functionality [12]. Therefore, pro-immunogenic maturation needs to be considered as a complex process that, in addition to mechanisms directly promoting T cell priming, also actively extinguishes specific pro-tolerogenic functions of DCs [50,51].

## 3. Activation of cDCs through Direct Sensing of Microbial Components and Other Inflammatory Molecules

**Figure 1:**
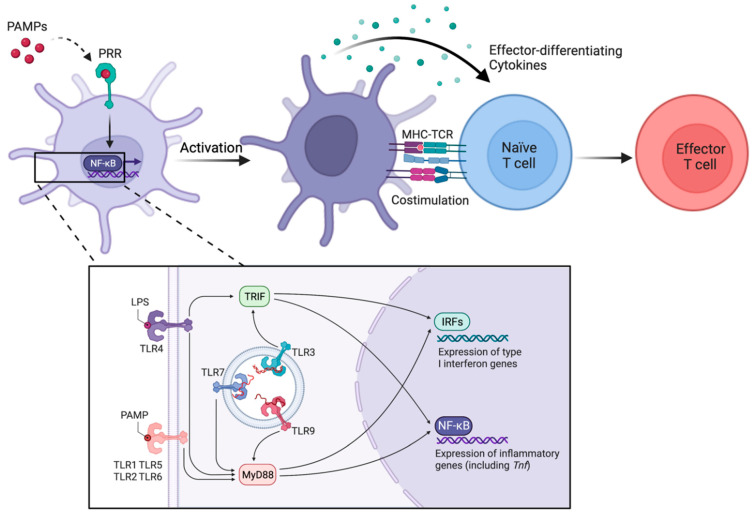
Activation of cDCs by microbial components enables effector T cell priming.

The direct activation of cDCs constitutes the critical initial step during the maturation process triggered by microbial components and is dependent on evolutionarily preserved PRRs that recognize various conserved PAMPs from a plethora of microorganisms [52]. These PRRs include toll-like receptors (TLRs), retinoic-acid-inducible gene (RIG)-like helicases (RLHs), and nucleotide-binding domain and leucine-rich repeat-containing molecules (NLRs) [53]. In addition to sensing extrinsic threats, cDCs can detect a variety of intracellular nucleic acids through multiple TLRs located in endosomal compartments within the cytosol, such as TLR3 (double-stranded [ds]RNA), TLR7 and TLR8 (single-stranded [ss]RNA), and TLR9 (CpG-DNA) [52]. The binding of TLRs to their ligands results in the activation of signaling cascades and the subsequent cellular responses and expression of inflammatory genes that are initiated by various adaptor molecules, including myeloid differentiation primary response gene 88 (MyD88) and toll-like receptor adaptor molecule 1 (TICAM1 or TRIF) [54]. All TLRs, except TLR3, signal through MyD88, while only TLR3 and TLR4 (which recognizes bacterial LPS) signal through TRIF [54]. cDCs also express the cytoplasmic dsRNA sensors retinoic acid-inducible gene I (RIG-I) and melanoma differentiation antigen 5 (MDA5), which signal through the adaptor molecule mitochondrial anti-viral signaling proteins (MAVS) [55,56,57]. Both the TLR and RIG-I/MDA5 signaling pathways intersect at the level of interferon regulatory factors (IRFs) and nuclear factor kappa-light-chain-enhancer of activated B cells (NF-κB), leading to transcriptional changes that result in the activation of cDCs and the subsequent production of cytokines and interferons [54,57]. Additional studies found that maturation induced by LPS and other PAMPs enhances in individual cDCs the functions of the inflammasome and increases the expression of major histocompatibility complexes (MHCs) as well as multiple co-stimulatory ligands, cytokines, and other molecules that collectively enable pro-immunogenic priming of effector T cells (Figure 1) [23,24,52,58].

In addition to PAMPs, cDCs can also be activated by damage-associated molecular patterns (DAMPs), which are endogenous molecules released from stressed, injured, or dying cells [15]. These molecules can bind to PRRs, including several TLRs, expressed by cDCs and activate them by inducing a variety of signaling pathways, including the NF-κB and mitogen-activated protein kinase (MAPK) pathways, ultimately leading to the upregulation of co-stimulatory molecules, cytokines, and chemokines that promote T cell activation [59]. One of the major PRRs involved in DAMP-mediated DC activation is TLR4, which recognizes several DAMPs, including high-mobility group box 1 (HMGB1) and heat shock proteins (HSPs) [60,61,62]. Similar to PAMPs, TLR4 engagement by DAMPs leads to the recruitment of adaptor molecules, including MyD88 and TRIF, which activate downstream signaling cascades, such as the NF-κB and MAPK pathways [63]. In addition to TLR4, other PRRs, including intracellular NOD-like receptors (NLRs) and C-type lectin receptors (CLRs), are also involved in DAMP-mediated DC activation. For example, NLRP3 inflammasome activation by DAMPs commonly produced by necrotic cells, such as uric acid crystals and ATP, leads to the production of interleukin (IL)-1β and IL-18 [64]. CLRs, such as Dectin-1 and DC-SIGN, can also recognize other DAMPs, such HSPs, and activate downstream signaling pathways that also promote cDC activation [65]. Additionally, Clec9A (also known as DNGR-1) is another CLR that is expressed specifically by cDC1s in both mice and humans and binds to exposed actin filaments, resulting in spleen-associated tyrosine kinase (SYK) and nicotinamide adenine dinucleotide phosphate (NADPH) oxidase activation and ultimately the cross-presentation of exogenous antigens [66,67]. Additionally, some of the specialization of cDC subsets can be partially explained by the differential expression of PRRs, such as TLRs and CLRs. For example, both mouse and human cDC1s express high levels of TLR3 and Clec9A, which contributes to their key role in both anti-tumor and anti-viral immune responses [15]. Overall, the activation of cDCs by both pathogen-associated and danger-associated molecules results in molecular re-programming that ultimately leads to the specific priming of T cells through the increased expression of effector-differentiating cytokines and co-stimulatory molecules, in addition to other changes that will be discussed further below.

Conventional dendritic cells (cDCs) express pattern recognition receptors (PRRs) that can sense pathogen-associated molecular patterns (PAMPs), which are conserved components of various classes of microbes and include the toll-like receptors (TLRs). Upon PRR signaling through pro-inflammatory pathways mediated by adaptor proteins, such as TRIF and MyD88, the expression of inflammatory genes, including type I interferons and tumor necrosis factor-alpha (*Tnf*), is mediated by transcription factors NF-κB and interferon regulatory transcription factors (IRFs). Furthermore, cDCs become activated through this process and express higher levels of co-stimulatory molecules, effector-differentiation cytokines, and major histocompatibility complexes (MHC), which are recognized by antigen-specific T cell receptors (TCR). These mechanisms work in concert to enable the pro-immunogenic priming of effector T cells by activated cDCs.

## 4. Amplification of cDC Maturation Process through Endogenous Secondary Mediators

Immunomodulatory mediators, including pro-inflammatory cytokines produced by cDCs, play an important role in T cell priming and skewing of their specific differentiation patterns [11,68]. Initially, the primary targets of cytokines, such as type I interferon (IFN-I) and tumor necrosis factor-alpha (TNF-α), that are released by cDCs upon their initial activation were thought to target other immune cells, and the role of these cytokines in the indirect activation of cDCs was not appreciated. These inflammatory cytokines were found to be insufficient for full dendritic cell activation and the promotion of T cell helper and cytotoxic functions [69,70]. However, other studies found that IFN-I enhanced the antibody response and induced isotype switching when cDCs were the only cell type responding to IFN-I [71]. Furthermore, the induction of efficient cross-priming during infection with lymphocytic choriomeningitis virus was found to be dependent on IFN-I [72]. Consistent with these observations, following stimulation with poly (I:C), a model TLR ligand, IFNs were found to dominate transcriptional changes in cDCs and were required for the upregulation of all (including metabolic) pathways associated with the maturation of cDCs and their increased immunogenicity [73]. Moreover, the IFN-I receptor and its STAT1-dependent signaling are essential for the maturation of cDCs and the development of CD4^+^ immunity [74]. These results contributed to the subsequent uncovering of quorum sensing of IFN-I by cDCs as a prerequisite for the effective activation of a cDC population in response to the original TLR stimulation [75]. Additionally supporting this key role of IFN-I in amplifying the responses by cDCs, the transcriptional profiling of intra-tumoral DCs within regressor tumors revealed an activation state of cDC2s characterized by the expression of interferon (IFN)-stimulated genes (ISGs) comparable to similar patterns found in cDC1s [76]. Furthermore, type III interferon (IFN-λ) signaling in cDCs is also critical for the development of protective CD8^+^ T cell responses [77].

In addition to the key role of IFN-I, TNF-α has also been established to mediate the maturation process of cDCs [69,71,75,78,79,80,81,82,83,84,85]. TNF-α, whose production is rapidly upregulated in response to extrinsic stimuli, such as house dust mites (HDMs), in combination with LPS sensitization, subsequently induces the expression of the transcription factor T-bet in maturing cDCs [78]. Additional research suggested that TNF-α signaling might affect cDC maturation both directly as well as indirectly through its effects on T cells [86]. The activated T cells can supply additional signals, such as through the CD40-CD40L axis, to further augment maturation by utilizing a positive feedback mechanism [87,88]. However, such signals are insufficient to trigger the maturation process in the absence of the innate stimulus [89,90]. Future research will further elucidate the impacts of these signals on cDC maturation and the resulting T cell responses.

An additional soluble secondary mediator involved in the maturation of cDCs is IL-1β. IL-1β is a pro-inflammatory cytokine that is produced by a variety of immune (including cDCs) and non-immune cells in response to infection, injury, or stress [91]. As mentioned earlier, NLRP3 activation results in the production of IL-1β, which can then activate DCs by binding to its receptor, IL-1R, on the surface [92]. This interaction leads to the recruitment of adaptor molecules, such as MyD88 and IL-1 receptor-associated kinases (IRAKs), which activate downstream signaling pathways, including the NF-κB and MAPK pathways [93]. These pathways ultimately lead to the upregulation of co-stimulatory molecules and the production of cytokines and chemokines that promote T cell activation [91]. Overall, crucial immunomediators released by activated cDCs enhance the propagation of the maturation process throughout the cDC populations, therefore amplifying the impact of the initial innate maturation stimulus.

## 5. Ablation of cDCs to Enhance and Terminate Immune Priming

After mature cDCs complete the priming of T cells, the death of cDCs may be beneficial to prevent excessive immune activation [94,95]. Initially, such death at the conclusion of the maturation process was primarily attributed to the elimination of cDCs by cytotoxic and other T cells through perforin-dependent mechanisms [96,97,98,99]. However, further experiments elucidated that the death of mature cDCs resulted from intrinsic mechanisms mediated by pro-apoptotic proteins, including BH3-only proteins, as a result of TLR3 and IFN-I signaling [100,101]. Nevertheless, other results also suggested a divergence of the mechanisms responsible for the activation-induced apoptosis of mature cDCs and other pathways that control the functional immunogenicity of cDCs during viral infection [102]. In contrast to undergoing a programmed cell death following the maturation process, a portion of cDCs also die shortly after initial activation [103,104,105]. This is consistent with other studies that reported an LPS-induced death and a reduced number of cDCs, especially among the CD8α^+^ subset. Such an early death of the activated cDCs was usually attributed to the mechanisms regulating T cell priming while also serving as a potential source of antigenic materials [25,106,107,108]. Similarly, the functions of glucocorticoids may induce the death of cDCs, including the loss of CD8α^+^ cDCs in a model of sepsis [109,110]. However, more recent studies uncovered relevant molecular mechanisms that selectively suppress the pyroptotic death of maturing cDCs, consequently maintaining such immunogenic cDCs and their immune functions [111,112]. Therefore, the death of mature cDCs emerges as a necessary process in order to avoid excessive T cell responses, but this death also needs to be tightly regulated to prevent adverse negative regulation of the maturation process.

In addition to the above-described roles of immunomodulators that limit the numbers of pro-immunogenic activated cDCs, recent results have revealed the key role of TNF-α in the ablation of cDCs with specific tolerogenic functions [51]. TNF-α is sensed through two different receptors, tumor necrosis factor receptor 1 (TNFR1) and TNFR2, whose specific expression and functions differ depending on the cell type in which they are expressed. While both receptors can respond to TNF-α bound to a membrane, only TNFR1 can proficiently respond to TNF-α in its soluble form [113]. TNFR1 is expressed by nearly all cell types, but TNFR2 expression is restricted to regulatory T cells, myeloid cells, glial cells, and certain endothelial cells and can additionally be induced in certain T and B cell subsets, epithelial cells, and fibroblasts [113]. Both receptors can induce pro-inflammatory pathways, such as the NF-κB pathway [113]. In contrast, TNFR1 additionally induces necroptotic and apoptotic cell death through complex processes dependent on receptor-interacting serine/threonine-protein kinase 3 (RIPK3) and caspase 8, respectively [113]. These diverse signaling modalities mediated by TNF-α underscore the pleiotropic outcomes of the sensing of this cytokine by cDCs. Specifically, among all the cDCs, the highest expression of TNFR1 characterizes tolerogenic cDCs [51]. These tolerogenic cDCs that belong to the cDC1 subset are characterized by the elevated expression of a key tolerance-promoting molecule, BTLA, and are, therefore, referred to as BTLA^hi^ cDC1 [7,14]. Through its interactions with herpesvirus entry mediator (HVEM), which is expressed in naïve T cells, BTLA facilitates the efficient conversion of pTreg cells upon the presentation of antigen-MHC II complexes by BTLA^hi^ cDC1s [7,11,12]. In T cells, HVEM signaling increases the surface expression of CD5 [7]. CD5 then reduces the functions of the mammalian target of rapamycin (mTOR) and promotes the conversion of naïve T cells to pTreg cells by decreasing their sensitivity to pro-inflammatory cytokines [114]. The BTLA^hi^ cDC1s are present in peripheral lymphoid organs, including the spleen and LNs and are, therefore, ideally positioned to present to T cells self-antigens, including those obtained from apoptotic materials [17,18,31,115,116,117]. The cDC-induced pTreg cells crucially regulate various immune and autoimmune responses [9,10,12,118,119,120]. These BTLA^hi^ cDC1s are directly killed in response to TNF-α binding to TNFR1 and the resulting RIPK3 and caspase 8 signaling [51]. This selective ablation of the tolerogenic BTLA^hi^ cDC1 population in mice was observed in response to both recombinant TNF-α administered without any other specific stimulus as well as endogenous TNF-α generated in vivo in response to treatment with LPS [51].

The antigen-specific pTreg cells induced by BTLA^hi^ cDC1s prevent effector T cells from achieving their immune and autoimmune competence under pro-inflammatory conditions [7,9]. Therefore, instead of serving as a direct feedback mechanism that restricts the immune response that is already in process, the rapid death of tolerogenic BTLA^hi^ cDCs can facilitate antigen-specific responses by removing these well-established immunosuppressive functions of pTreg cells [8,9,32]. Furthermore, the prevention of de novo generation of pTreg cells skews the balance of T cell commitment to favor the early effector T cells that normally accompany pTreg cell conversion [121]. Because TNF-α is produced by cDCs and other cells in response to a pro-inflammatory stimulus, such as LPS [51,122], the increase in TNF-α rapidly amplifies the loss of a tolerogenic cDC population and essentially removes the capacity to induce new antigen-specific regulatory T cells at the onset of a maturation process [51]. Anti-TNF-α therapies have been successfully introduced for the treatment of several autoimmune diseases, including rheumatoid arthritis and Crohn’s disease, possibly suggesting the role of the TNF-α-mediated ablation of tolerogenic cDCs in these autoimmune responses [123]. However, the multiple outcomes of signaling through TNFR1 and TNFR2 on cDCs and T cells complicate the analysis of the direct impacts of TNF-α-blocking approaches on specific autoimmune responses. For example, the anti-TNF-α therapies were unsuccessful, if not detrimental, in the case of multiple sclerosis (MS) [50,123,124]. Instead, combining the specific delivery of neuronal antigens to tolerogenic cDCs [118,120,124] with specific therapeutic strategies to avoid the loss of cDCs under disease conditions might present a clinically relevant opportunity for the treatment of MS through re-establishing tolerance. The ablation of cDCs occurring as part of the maturation process raises possible concerns about the long-term effects of such cDC loss. The enduring sequelae of infections may bring about a more permanent transformation of the murine cDC populations at specific anatomical locations [125]. However, as mentioned earlier, the life span of cDCs in vivo is much shorter than that of T cells and other long-lived immune cells [21]. Therefore, following an acute decline caused by TNF-α, the numbers of BTLA^hi^ cDCs return to their homeostatic baseline in less than a week after being replenished from their bone marrow precursors [51]. This cycle of life and death of tolerogenic cDCs allows for the abrupt termination of their functions to facilitate the propagation of immune responses while ensuring the continuation of their tolerogenic functions [50]. A clearly defined homeostatic baseline and responsivity not only to innate but also other signals also distinguish systemic cDCs from other antigen-presenting cells (APCs) present at anatomical barriers, such as in the intestines, that are constantly exposed to signals derived from the microbiota [20].

## 6. Concluding Remarks

**Figure 2:**
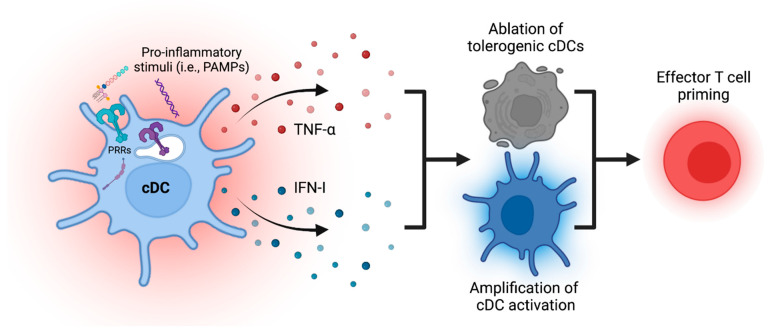
Production of secondary mediators by activated cDCs enhances immune priming.

In contrast to the concept of maturation as a one-step process, the emerging paradigm postulates diverse processes that all collectively contribute to the maturation process. This also underscores a division of labor amongst cDCs found at the anatomical barriers and those present systemically. While at the barriers, cDCs are constantly exposed to commensal microbiota and, therefore, specific exogenous signals, systemic cDCs serve as early sentinels of microbial infections and become activated in response to microbe-produced factors, such as LPS. To ensure an adequate immune response, the initial activation of cDCs is rapidly amplified to achieve immunocompetence by cDCs that were not immediately exposed to the initial infection. The specific ablation of tolerogenic cDCs helps to facilitate the ensuing effector response. The amplification of such tolerogenic cDC ablation, resulting in the rapid, albeit transient, removal of the tolerogenic population, is of particular biological significance due to the dominant nature of immunoregulatory mechanisms (Figure 2). Conversely, the subsequent ablation of highly immunogenic cDCs helps to terminate the priming of T cells to avoid excessive immunogenicity. The understanding of these complex maturation processes will have an impact on immunotherapies and vaccine design. Future studies could have particularly significant implications clinically for patients with numerous chronic autoimmune and inflammatory diseases that are distinguished by the excessive production of TNF-α but for which current anti-TNF-α treatments have been unsuccessful, such as MS.

The sensing of pro-inflammatory stimuli, such as pathogen-associated molecular patterns (PAMPs) and pathogen recognition receptors (PRRs), leads to the activation of some conventional dendritic cells (cDCs) and the subsequent production of secondary mediators, including tumor necrosis factor-alpha (TNF-α) and type I interferons (IFN-I). These mediators can be sensed by other cDCs and lead to the ablation of specific tolerogenic cDCs and the activation of other cDCs that do not necessarily sense the initial stimulus. Together, these changes in the cDC population lead to enhanced effector T cell priming while limiting the induction of tolerogenic mechanisms.

## Data Availability

Not applicable.
